# Assessing Nephrological Competence among Geriatricians: A Proof of Concept Internet Survey

**DOI:** 10.1371/journal.pone.0141388

**Published:** 2015-11-03

**Authors:** Raffaele Antonelli Incalzi, Filippo Aucella, Dario Leosco, Giuliano Brunori, Michela Dalmartello, Giuseppe Paolisso

**Affiliations:** 1 Dept of Geriatrics, Campus Bio-Medico University, Rome, Italy; 2 Dept of Nephrology and Dialysis, IRCCS Casa Sollievo della Sofferenza Hospital, San Giovanni Rotondo, Foggia, Italy; 3 Dept of Translational Medical Science, University of Naples Federico II, Napoli, Italy; 4 Dept of Nephrology and Dialysis, S. Chiara Hospital, Trento, Italy; 5 Dept. of Health and Social Service, Provincia Autonoma di Trento, Italy; 6 Department of Medical, Surgical, Neurological, Aging and Metabolic Sciences, Second University of Naples, Napoli, Italy; University of Naples Federico II, ITALY

## Abstract

Chronic kidney disease (CKD) is highly prevalent in the elderly and negatively impacts survival and health status. Thus, nephrological competence is mandatory for a skilled geriatrician. The present study aimed to assess nephrological competence in a sample of geriatricians recruited through a web survey. To this aim, a 12-items questionnaire was produced by an expert panel of nephrologists and geriatricians and was available online for members of the Italian Society of Gerontology and Geriatrics (SIGG). Two-hundred-eighty-seven geriatricians volunteered to fill in the questionnaire. The majority of them indirectly estimated the glomerular filtration rate (GFR) using mainly the Cockroft-Gault (C-G) formula. Selected nephrological exams, such as urinary Na and serum D-vitamin measurements, did not qualify as routine exams although the majority of geriatricians supplemented their patients with fat-soluble secosteroids. Ten percent of geriatricians asked for nephrological consultation only for stage 5 CKD patients and 30,9% only for stage 4 or 5. Erythropoietin supplementation was common practice for the majority of geriatricians, while only one third of them systematically used a procedure intended to prevent the contrast induced nephropathy (CIN). Finally, an alleged 50% adherence to the international guidelines for the management of CKD patients emerged from the questionnaire. Overall, results from this survey strongly recommend promoting nephrological education among geriatricians. Didactic standards for in training geriatricians need to be updated and the cooperation between geriatrics and nephrological societies promoted.

## Introduction

Chronic kidney disease (CKD) is a typical age-related condition due to physiological decline in glomerular filtration rate (GFR), progressive increase in survival and, mostly, increased burden of multimorbidity. It dramatically impacts survival and other major outcomes, such as health status, in the general and in many selected populations [1–5]. Furthermore, it remains frequently unrecognized [6], and this adversely affects the provision of care, e. g. through overdosage of renally cleared drugs or improper use of intravenous contrast media [7]. Thus, being aware of nephrological problems is mandatory for a skilled geriatrician.

The objective of the present study was to assess nephrological competence in a sample of geriatricians recruited through a web survey.

## Methods

A series of 12 questions assessing nephrological competence was produced on the basis of an expert opinion review of nephrological problems of prominent interest for the practicing geriatrician made by two geriatricians and two nephrologists (Table 1). Thus, the items were fully oriented to clinical practice. The full set of questions intended to explore the every day clinical behavior and not the nephrological culture, if not indirectly. Furthermore, it focuses on items which are of primary clinical interest, but are not deeply rooted into routine geriatric practice. Accordingly, we did not enquire about health status assessment or prevention and detection of adverse drug reactions given that these items, of primary nephrological importance, are key parts of the comprehensive geriatric assessment.

**Table 1:** The Nephrological Questionnaire for Geriatricians.

1	Do you systematically assess GFR?				
	Yes, 71.5%				
2	In the event of a confirmatory answer to question 1: through				
	Creatinine clearance: 8%	MDRD: 45.5%	C-G: 65%		
3	Do you routinely check the following parameters?				
	Urine[Na]: 25%	Urine[N]: 19%	Urine[P]: 16%	S-OH VitD: 30%	
4	Do you routinely perform 24 hour urine collection?				
	Yes, 69%				
5	At which CKD stage do you refer your patient to the nephrologist?				
	CKD 2: 5.4%	CKD 3a: 16.2%	CKD 3b: 34.7%	CKD 4: 32.9%	CKD 5: 10.8%
6	Do you think that EPO supplementation is useful to your patients?				
	Yes, 90.3%				
7	Do you usually prescribe D vitamin to your patients?				
	Yes, 90.3%				
8	In the event of a confirmatory answer to question 7, which D vitamin do you prescribe?				
	Cholecalciferol: 61.4%	25(OH)D3 calcidiol: 24.1%	Calcitriol: 23.1%	Other: 1.4%	
9	Do you usually check the albumin to creatinine ratio on spot urine sample?				
	Yes, 12%				
10	Do you usually order renal ultrasound scan?				
	Yes, 74.4%				
11	Do you use a standardized protocol to prevent the contrast induced nephropathy?				
	31%				
12	How do you rate your adherence to nephrological guidelines (from 1 to 5)?				
	1) 3.1%	2) 12%	3) 67.4%	4) 26.4%	5) 1.2%

GFR, Glomerular Filtration Rate; MDRD, Mediterranean Diet Renal Disease; C-G, Cokroft-Gault; S-OH VitD, Serum-OH Vitamin D; CKD, Chronic Kidney Disease; EPO, Eritropoietin.

The questionnaire was available on line for members of the Italian Society of Gerontology and Geriatrics (SIGG). This study was designed as a survey of nephrological competence among geriatricians; they could become aware of the survey by reading the News section of the SIGG website (www.sigg.it), but they were not formally informed of it. Thus, all participants contributed on a voluntary basis and released information that was taken anonymous. In the introduction to the questionnaire responders were recommended to focus exclusively on their usual nephrological practice. Accordingly, questions like the number 4, which explores the use of 24 hours urine collection, a procedure very useful also in non nephrological conditions, had to be intended in the context of nephrological practice.

The study did not involve patients or patient-originated data and as such, it did not require formal approval by Institutional Review Boards.

In the era of internet, the SIGG-SIN joint commission intended to explore the possibility that a “low cost” e survey could provide a potentially important clinical information. Accordingly, no formal announcement or advertising was used to promote the questionnaire on the assumption that the simple access to SIGG website and the personal sensibility of readers would have make such a survey feasible. We could not find any comparable experience of e survey, which makes the present one a proof of concept low cost e survey.

Questionnaires were analyzed by descriptive statistics.

## Results

Two-hundred-eighty-seven geriatricians out of the 1601 SIGG members filled in the questionnaire. Individual questions and the responses are reported in Table 1 and S1 Dataset.

## Discussion

Geriatricians were aware of how CKD is relevant to the health of their patients and then, to their practice. The majority of them indirectly estimated the GFR. The frequent measurement of the creatinine clearance likely reflects the fact that a consistent proportion of geriatricians cared for disabled and catheterized patients. On the other hand, the more frequent use of the Cockroft-Gault (C-G) formula with regard to the Modification of Diet in Renal Disease (MDRD) formula might be explained by the setting: in frail and sarcopenic patients the C-G formula seems preferable to the MDRD [8]. At variance from the GFR, the albumin to creatinine ratio (ACR) was systematically checked only by about one third of geriatricians. This is alarming because an abnormal ACR with normal GFR heralds renal failure not only in adult diabetics, but also in hypertensive patients [9], whereas selected nutritional and pharmacological interventions can efficaciously slow this evolution [10]. Furthermore, proteinuria has recently been shown to predict end-stage renal disease (ESRD) also in the elderly, especially in the early stages of CKD [11].

Overall, estimated GFR (eGFR) and albuminuria thresholds, according to Kidney Disease Improving Global Outcomes guidelines [12], are deemed appropriate to define CKD across all age subgroups. Thus, eGFR and albuminuria should complement traditional risk factors in the cardiovascular risk charts for the general population [13]. Interestingly, in a meta-analysis involving 2,051,244 participants from 33 general population or high cardiovascular risk cohorts and 13 CKD cohorts, depressed eGFR was more strictly associated with excess mortality in older than in middle-aged adults, whereas the risk of ESRD similarly increased in all age groups with either eGFR decline below 60 mL/min/1.73 m2 or increasing albuminuria [12,14].

While it is not surprising that selected nephrological exams, such as the measurement of urinary Na and N, are out of the geriatric routine, the infrequent screening for D vitamin deficiency deserves consideration. Given that the majority of geriatricians supplemented their patients with D vitamin, it should be speculated that most of them judged D vitamin measurement superfluous. However, neither D vitamin deficiency can be assumed a priori in all CKD patients nor systematic supplementation seems a motivated strategy. Indeed, the threshold of eGFR<30 ml/min/1.73 m^2^ seems to mark the need for D vitamin supplementation [15], but elderly people are highly exposed to the risk of vitamin deficit even in the absence of CKD.

The fact that 10,5% of geriatricians asked for nephrological consultation only for stage 5 CKD patients and 30,9% only for stage 4 or 5 confirms findings pertaining to a tertiary geriatric outpatient clinic [16]. Paradoxically, comorbidity, i. e. an index of complexity of the health status, has been reported to characterize patients for whom general practitioners did not ask for nephrologic consultation [17]. These findings are of concern. Indeed, patients with severe CKD require a multidisciplinary approach having as central the contribution of the nephrologist. Recently published international and Italian guidelines [18,19] state that the referral to specialist kidney care services is recommended for CKD people with GFR <30 ml/min/1.73 m^2^ (GFR categories G4-G5) or a consistent finding of significant albuminuria. However, if albuminuria is a stable isolated finding, advice from specialist services may substitute for formal referral depending upon the available health-care facilities. However, more recently a significant rise in ESRD and mortality risks has been observed in patients with stage 3b CKD who were not referred to a nephrologist [20]. Furthermore, in older patients on nephrology care, the risk of ESRD prevailed over that of death even when eGFR was only moderately impaired [11].

Geriatricians were fully aware of the need of erythropoietin analogues and D vitamin supplementation. However, only about 30% of the geriatricians used calcitriol as D vitamin supplement for CKD patients. Furthermore, only one third of geriatricians systematically used a procedure intended to prevent the contrast induced nephropathy (CIN). This is alarming because age and multimorbidity are the main risk factors for CIN behind depressed GFR itself [21]. Furthermore, selected interventions and precautions have the potential for decreasing the risk of CIN [22]. Analogously, an alleged 50% adherence to the international guidelines for the management of CKD patients cannot be considered a satisfactory result. Interestingly, comparable figures define the adherence to guidelines for the care of a highly prevalent chronic condition such as congestive heart failure (CHF) [23]. Poor adherence recognizes many reasons, at least as we can infer from the CHF model [24]. However, it is out of doubt that adherence is associated with better clinical outcomes, at least in CHF patients [25]. In our probands, adherence might be even worse than reported. Indeed, selected procedures which are strongly recommended by the most common nephrological guidelines, such as calcitriol supplementation in advanced CKD or measurement of AUC, were common practice for a fraction of responders which was lower than that reporting adherence to guidelines. Furthermore, there was no difference in the routine use of selected exams expected to be frequently ordered, e g urine Na, and other, e g, urine Nitrogen, which are less commonly indicated (answer to question 3). Thus, this survey points at a methodological problem which needs to be considered when interpreting “summary questions” such as that exploring the adherence to guidelines.

This survey has many and important limitations. First, it was addressed to geriatricians selected on the basis of their familiarity with internet and interest in the topic. Thus, the sample cannot be considered representative of the typical geriatrician: this recruitment procedure likely guarantees that the most motivated geriatricians were interviewed. Thus, the real life situation is likely worse than that observed. Collaterally, the low rate of responders, 18% of potential responders, is itself an useful finding as it shows that some form of active recruitment is needed to gather a representative information. Overconfidence in the e communication resulted in a low participation survey. Second, we gathered no information on the workplace of responders: selected practices, e. g. CIN prevention, might be setting dependent, more familiar to hospital based geriatricians. Third, the geriatricians were asked whether they usually adhere to current nephrological guidelines in their daily practice; this question might be too generic because the source of the guidelines is not specified. Forth, we asked whether GFR was indirectly estimated through the MDRD or the C-G formula, but not through the CK-EPI or the BIS formula. At the time of the planning of the study formulas as the CKD-EPI and the BIS were not so commonly used and, thus, were excluded from the questionnaire. We did not collect information about age and professional role of our probands. This prevents us from interpreting to which kind of geriatrician the collected information applies. Finally, it is important to underline that our results are referring to a selected Italian experience.

Despite these limitations, this survey suggests that nephrological education should be promoted among geriatricians. Indeed, nephrological training is not included in the geriatric curriculum recommended by the Italian Minister of Education: the availability of the urologic, but not of the nephrological service is deemed essential for the training in Geriatrics [26]. Furthermore, only from 2013 the SIGG has regularly promoted the nephrological culture by a joint SIN-SIGG session in the yearly SIGG congress and through selected local meetings. Thus, didactic standards for in training geriatricians need to be updated and the cooperation between geriatrics and nephrological societies promoted. Collaterally, this experience shows that, despite the spread of e communication, a sort of spontaneous survey cannot be recommended, at least if the overall category of specialists represents its target. Some form of advertising is needed to collect a representative sample of the target category.

## Supporting Information

S1 Dataset Questionnaire Responses Dataset. (XLS)
